# Hexaaqua­nickel(II) bis­[4-(2-hydroxy­benzyl­ideneamino)benzene­sulfonate]

**DOI:** 10.1107/S1600536808015262

**Published:** 2008-06-07

**Authors:** Xi-Shi Tai, Jun Xu, Yi-Min Feng, Zu-Pei Liang

**Affiliations:** a Department of Chemistry and Chemical Engineering, Weifang University, Weifang 261061, People’s Republic of China; b Weifang Institute of Supervision and Inspection, on Product Quality, Weifang 261031, People’s Republic of China

## Abstract

In the title compound, [Ni(H_2_O)_6_](C_(C_13__H_10_NO_4_S)_2_, the nickel(II) atom, lying on a center of symmetry, is six-coordinated by six aqua O-atom donors. The dihedral angle between the two benzene rings is 33.1 (3)°. The crystal structure is stabilized by aqua–anion O—H⋯O hydrogen bonds. Intra­molecular O—H⋯N and C—H⋯O hydrogen-bonding inter­actions occur in the anion.

## Related literature

For related literature, see: Tai & Feng (2008 ▶); Tai *et al.* (2003 ▶, 2008 ▶); Tai, Yin & Feng (2007 ▶); Tai, Yin & Hao (2007 ▶); Tai, Yin, Feng & Kong (2007 ▶ ▶ ▶); Wang *et al.* (2007 ▶).
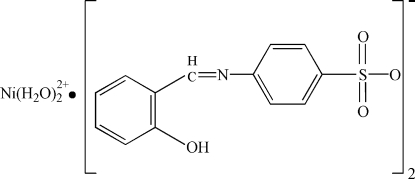


## Experimental

### 

#### Crystal data

[Ni(H_2_O)_6_](C_13_H_10_NO_4_S)_2_
*M* *_r_* = 719.37
Monoclinic, 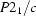

*a* = 6.3047 (6) Å
*b* = 35.193 (3) Å
*c* = 9.3536 (10) Å
β = 131.822 (2)°
*V* = 1546.6 (3) Å^3^
*Z* = 2
Mo *K*α radiation
μ = 0.83 mm^−1^
*T* = 298 (2) K
0.43 × 0.38 × 0.25 mm

#### Data collection

Bruker SMART CCD area-detector diffractometer
Absorption correction: multi-scan (*SADABS*; Bruker, 2000 ▶) *T*_min_ = 0.716, *T*_max_ = 0.819
7465 measured reflections
2661 independent reflections
2428 reflections with *I* > 2σ(*I*)
*R*_int_ = 0.032

#### Refinement

*R*[*F* ^2^ > 2σ(*F* ^2^)] = 0.067
*wR*(*F* ^2^) = 0.137
*S* = 1.28
2661 reflections
205 parameters
H-atom parameters constrained
Δρ_max_ = 0.32 e Å^−3^
Δρ_min_ = −1.00 e Å^−3^

### 

Data collection: *SMART* (Bruker, 2000 ▶); cell refinement: *SAINT* (Bruker, 2000 ▶); data reduction: *SAINT*; program(s) used to solve structure: *SHELXS97* (Sheldrick, 2008 ▶); program(s) used to refine structure: *SHELXL97* (Sheldrick, 2008 ▶); molecular graphics: *SHELXTL* (Sheldrick, 2008 ▶); software used to prepare material for publication: *SHELXTL*.

## Supplementary Material

Crystal structure: contains datablocks global, I. DOI: 10.1107/S1600536808015262/at2568sup1.cif

Structure factors: contains datablocks I. DOI: 10.1107/S1600536808015262/at2568Isup2.hkl

Additional supplementary materials:  crystallographic information; 3D view; checkCIF report

## Figures and Tables

**Table 1:** Hydrogen-bond geometry (Å, °)

*D*—H⋯*A*	*D*—H	H⋯*A*	*D*⋯*A*	*D*—H⋯*A*
O4—H4⋯N1	0.82	1.86	2.595 (9)	148
O5—H5*A*⋯O1^i^	0.85	1.96	2.737 (6)	151
O5—H5*B*⋯O2^ii^	0.85	1.98	2.751 (6)	150
O6—H6*A*⋯O3^i^	0.85	1.95	2.764 (7)	161
O6—H6*B*⋯O1^iii^	0.85	1.97	2.768 (8)	156
O7—H7*A*⋯O2	0.85	2.00	2.757 (8)	148
O7—H7*B*⋯O3^ii^	0.85	2.00	2.769 (7)	150
C2—H2⋯O3	0.93	2.56	2.917 (7)	104

Symmetry codes: (i) 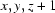; (ii) 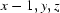; (iii) 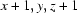.

**Fig. 1.**
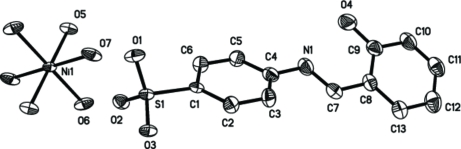
The molecular structure of (I) showing 30% displacement ellipsoids.

## supplementary crystallographic information

### Comment

As part of our ongoing studies of the coordination chemistry of Schiffbase ligands (Tai *et al.*, 2003; (Tai, Yin & Feng, 2007; Tai, Yin, Feng & Kong, 2007; Tai, Yin & Hao, 2007; Wang *et al.*, 2007; Tai *et al.*, 2008; Tai *et al.*, 2008), we now report the synthesis and structure of the title compound, (I), (Fig. 1).

In the molecule of (I), the nickel (II) center is six-coordinate with six O donor of H_2_O. The C7—N1 [1.288 (8) Å] is close to double-bond. Otherwise, the geometrical parameters for (I) are normal. The dihedral angle between the two benzene rings is 33.1°. The packing is stabilized by O_water_—H···O_anion_ hydrogen bonds. The intramolecular O—H···N and C—H···O hydrogen bonding interactions occur in the anion. (Table 1).

### Experimental

1 mmol of Ni(CH_3_COO)_2_.4H_2_O was added to a solution of salicylaldehyde-4-aminobenzene sulfonic acid (1 mmol) in 10 ml of 95% ethanol. The mixture was stirred for 2 h at refluxing temperature. Evaporating some ethanol, clear blocks of (I) were obtained after one weeks.

### Refinement

The H atoms were placed geometrically (C—H = 0.93 Å and O—H = 0.82-0.85Å) and refined as riding with *U*_iso_(H) =1.2 or 1.5*U*_eq_(carrier).

### Crystal data

**Table:**

[Ni(H_2_O)_6_](C_13_H_10_NO_4_S)_2_	*F*_000_ = 748
*M**_r_* = 719.37	*D*_x_ = 1.545 Mg m^−^^3^
Monoclinic, *P*2_1_/*c*	Mo *K*α radiation λ = 0.71073 Å
Hall symbol: -P 2ybc	Cell parameters from 4537 reflections
*a* = 6.3047 (6) Å	θ = 2.9–27.7º
*b* = 35.193 (3) Å	µ = 0.83 mm^−^^1^
*c* = 9.3536 (10) Å	*T* = 298 (2) K
β = 131.822 (2)º	Block, colourless
*V* = 1546.6 (3) Å^3^	0.43 × 0.38 × 0.25 mm
*Z* = 2	

### Data collection

**Table:**

Bruker SMART CCD area-detector diffractometer	2661 independent reflections
Radiation source: fine-focus sealed tube	2428 reflections with *I* > 2σ(*I*)
Monochromator: graphite	*R*_int_ = 0.032
*T* = 298(2) K	θ_max_ = 25.0º
φ and ω scans	θ_min_ = 2.3º
Absorption correction: multi-scan(SADABS; Bruker, 2000)	*h* = −7→5
*T*_min_ = 0.716, *T*_max_ = 0.819	*k* = −41→40
7465 measured reflections	*l* = −9→11

### Refinement

**Table:**

Refinement on *F*^2^	Secondary atom site location: difference Fourier map
Least-squares matrix: full	Hydrogen site location: inferred from neighbouring sites
*R*[*F*^2^ > 2σ(*F*^2^)] = 0.067	H-atom parameters constrained
*wR*(*F*^2^) = 0.137	*w* = 1/[σ^2^(*F*_o_^2^) + (0.0161*P*)^2^ + 4.8144*P*] where *P* = (*F*_o_^2^ + 2*F*_c_^2^)/3
*S* = 1.28	(Δ/σ)_max_ < 0.001
2661 reflections	Δρ_max_ = 0.32 e Å^−^^3^
205 parameters	Δρ_min_ = −1.00 e Å^−^^3^
Primary atom site location: structure-invariant direct methods	Extinction correction: none

### Special details

**Table:**

Geometry. All e.s.d.'s (except the e.s.d. in the dihedral angle between two l.s. planes) are estimated using the full covariance matrix. The cell e.s.d.'s are taken into account individually in the estimation of e.s.d.'s in distances, angles and torsion angles; correlations between e.s.d.'s in cell parameters are only used when they are defined by crystal symmetry. An approximate (isotropic) treatment of cell e.s.d.'s is used for estimating e.s.d.'s involving l.s. planes.
Refinement. Refinement of *F*^2^ against ALL reflections. The weighted *R*-factor *wR* and goodness of fit *S* are based on *F*^2^, conventional *R*-factors *R* are based on *F*, with *F* set to zero for negative *F*^2^. The threshold expression of *F*^2^ > σ(*F*^2^) is used only for calculating *R*-factors(gt) *etc*. and is not relevant to the choice of reflections for refinement. *R*-factors based on *F*^2^ are statistically about twice as large as those based on *F*, and *R*- factors based on ALL data will be even larger.

### Fractional atomic coordinates and isotropic or equivalent isotropic displacement parameters (Å^2^)

**Table:**

	*x*	*y*	*z*	*U*_iso_*/*U*_eq_	
Ni1	0.5000	1.0000	1.0000	0.0310 (2)	
N1	0.4029 (11)	0.76969 (14)	0.4874 (8)	0.0565 (13)	
O1	0.3354 (7)	0.95021 (11)	0.3248 (5)	0.0486 (10)	
O2	0.6843 (8)	0.94957 (11)	0.6726 (5)	0.0462 (9)	
O3	0.8302 (7)	0.93929 (10)	0.4933 (5)	0.0392 (8)	
O4	0.0669 (10)	0.71618 (13)	0.4256 (8)	0.0767 (14)	
H4	0.1257	0.7379	0.4400	0.115*	
O5	0.2072 (7)	0.97532 (11)	0.9978 (5)	0.0474 (9)	
H5A	0.2819	0.9621	1.0975	0.071*	
H5B	0.0852	0.9623	0.8972	0.071*	
O6	0.7945 (8)	0.96016 (12)	1.1929 (6)	0.0549 (11)	
H6A	0.8124	0.9592	1.2913	0.082*	
H6B	0.9559	0.9642	1.2273	0.082*	
O7	0.3791 (8)	0.96590 (13)	0.7796 (6)	0.0600 (12)	
H7A	0.4709	0.9703	0.7444	0.090*	
H7B	0.2009	0.9665	0.6838	0.090*	
S1	0.6046 (2)	0.93471 (4)	0.49681 (17)	0.0319 (3)	
C1	0.5566 (9)	0.88532 (14)	0.4986 (7)	0.0313 (10)	
C2	0.6556 (12)	0.86005 (16)	0.4406 (8)	0.0451 (13)	
H2	0.7524	0.8689	0.4039	0.054*	
C3	0.6082 (14)	0.82145 (16)	0.4382 (9)	0.0529 (15)	
H3	0.6758	0.8042	0.4012	0.063*	
C4	0.4623 (12)	0.80853 (16)	0.4899 (9)	0.0483 (14)	
C5	0.3614 (13)	0.83417 (16)	0.5454 (9)	0.0509 (15)	
H5	0.2614	0.8253	0.5795	0.061*	
C6	0.4079 (12)	0.87246 (15)	0.5503 (8)	0.0438 (13)	
H6	0.3404	0.8896	0.5878	0.053*	
C7	0.5721 (13)	0.74368 (17)	0.5168 (9)	0.0525 (15)	
H7	0.7384	0.7507	0.5446	0.063*	
C8	0.5084 (15)	0.70327 (16)	0.5074 (9)	0.0527 (14)	
C9	0.2544 (14)	0.69149 (18)	0.4569 (9)	0.0589 (17)	
C10	0.1982 (16)	0.65268 (18)	0.4407 (9)	0.0643 (18)	
H10	0.0295	0.6445	0.4068	0.077*	
C11	0.3845 (17)	0.6265 (2)	0.4733 (9)	0.068 (2)	
H11	0.3396	0.6008	0.4588	0.082*	
C12	0.6424 (19)	0.6376 (2)	0.5281 (11)	0.077 (2)	
H12	0.7726	0.6196	0.5549	0.092*	
C13	0.7008 (15)	0.67614 (18)	0.5417 (9)	0.0610 (17)	
H13	0.8689	0.6840	0.5739	0.073*	

### Atomic displacement parameters (Å^2^)

**Table:**

	*U*^11^	*U*^22^	*U*^33^	*U*^12^	*U*^13^	*U*^23^
Ni1	0.0214 (4)	0.0436 (5)	0.0287 (4)	−0.0007 (4)	0.0170 (4)	−0.0012 (4)
N1	0.060 (3)	0.044 (3)	0.061 (3)	−0.010 (2)	0.038 (3)	−0.004 (2)
O1	0.0284 (19)	0.064 (2)	0.048 (2)	0.0099 (18)	0.0234 (19)	0.0211 (19)
O2	0.042 (2)	0.056 (2)	0.051 (2)	−0.0096 (18)	0.035 (2)	−0.0105 (18)
O3	0.0269 (17)	0.055 (2)	0.041 (2)	−0.0029 (16)	0.0250 (16)	0.0003 (17)
O4	0.056 (3)	0.059 (3)	0.094 (4)	−0.008 (2)	0.042 (3)	0.002 (3)
O5	0.0309 (19)	0.074 (3)	0.035 (2)	−0.0104 (19)	0.0206 (17)	−0.0005 (19)
O6	0.038 (2)	0.082 (3)	0.055 (3)	0.018 (2)	0.035 (2)	0.025 (2)
O7	0.036 (2)	0.100 (3)	0.053 (3)	−0.023 (2)	0.033 (2)	−0.033 (2)
S1	0.0229 (6)	0.0420 (7)	0.0311 (6)	−0.0014 (5)	0.0181 (5)	0.0003 (5)
C1	0.021 (2)	0.041 (3)	0.027 (2)	−0.004 (2)	0.014 (2)	−0.002 (2)
C2	0.053 (3)	0.049 (3)	0.047 (3)	−0.003 (3)	0.039 (3)	−0.004 (3)
C3	0.066 (4)	0.046 (3)	0.054 (4)	−0.006 (3)	0.043 (3)	−0.012 (3)
C4	0.041 (3)	0.046 (3)	0.049 (3)	−0.007 (3)	0.026 (3)	0.001 (3)
C5	0.054 (4)	0.046 (3)	0.068 (4)	−0.004 (3)	0.047 (4)	0.001 (3)
C6	0.053 (3)	0.044 (3)	0.053 (3)	−0.004 (3)	0.043 (3)	0.001 (3)
C7	0.050 (4)	0.056 (4)	0.048 (4)	−0.014 (3)	0.031 (3)	−0.009 (3)
C8	0.059 (4)	0.045 (3)	0.047 (3)	−0.008 (3)	0.032 (3)	−0.008 (3)
C9	0.050 (4)	0.049 (4)	0.048 (4)	−0.003 (3)	0.021 (3)	0.002 (3)
C10	0.065 (4)	0.049 (4)	0.055 (4)	−0.014 (3)	0.030 (4)	−0.002 (3)
C11	0.082 (5)	0.050 (4)	0.051 (4)	−0.013 (4)	0.035 (4)	0.000 (3)
C12	0.098 (6)	0.049 (4)	0.079 (5)	0.000 (4)	0.057 (5)	0.002 (4)
C13	0.068 (4)	0.052 (4)	0.063 (4)	−0.007 (3)	0.044 (4)	−0.007 (3)

### Geometric parameters (Å, °)

**Table:**

Ni1—O5^i^	2.028 (3)	C1—C2	1.388 (7)
Ni1—O5	2.028 (3)	C2—C3	1.388 (8)
Ni1—O7^i^	2.043 (4)	C2—H2	0.9300
Ni1—O7	2.043 (4)	C3—C4	1.371 (8)
Ni1—O6	2.049 (4)	C3—H3	0.9300
Ni1—O6^i^	2.049 (4)	C4—C5	1.389 (8)
N1—C7	1.288 (8)	C5—C6	1.373 (8)
N1—C4	1.414 (7)	C5—H5	0.9300
O1—S1	1.458 (4)	C6—H6	0.9300
O2—S1	1.459 (4)	C7—C8	1.465 (8)
O3—S1	1.454 (3)	C7—H7	0.9300
O4—C9	1.333 (8)	C8—C9	1.397 (9)
O4—H4	0.8209	C8—C13	1.402 (9)
O5—H5A	0.8494	C9—C10	1.394 (8)
O5—H5B	0.8492	C10—C11	1.357 (10)
O6—H6A	0.8500	C10—H10	0.9300
O6—H6B	0.8498	C11—C12	1.396 (10)
O7—H7A	0.8497	C11—H11	0.9300
O7—H7B	0.8504	C12—C13	1.387 (9)
S1—C1	1.767 (5)	C12—H12	0.9300
C1—C6	1.387 (7)	C13—H13	0.9300
			
O5^i^—Ni1—O5	180.0 (2)	C3—C2—H2	120.5
O5^i^—Ni1—O7^i^	90.78 (15)	C1—C2—H2	120.5
O5—Ni1—O7^i^	89.22 (15)	C4—C3—C2	120.4 (5)
O5^i^—Ni1—O7	89.22 (15)	C4—C3—H3	119.8
O5—Ni1—O7	90.78 (15)	C2—C3—H3	119.8
O7^i^—Ni1—O7	180.000 (2)	C3—C4—C5	119.9 (5)
O5^i^—Ni1—O6	89.99 (15)	C3—C4—N1	123.2 (6)
O5—Ni1—O6	90.01 (15)	C5—C4—N1	116.9 (5)
O7^i^—Ni1—O6	90.23 (18)	C6—C5—C4	120.6 (5)
O7—Ni1—O6	89.77 (18)	C6—C5—H5	119.7
O5^i^—Ni1—O6^i^	90.01 (15)	C4—C5—H5	119.7
O5—Ni1—O6^i^	89.99 (15)	C5—C6—C1	119.1 (5)
O7^i^—Ni1—O6^i^	89.77 (18)	C5—C6—H6	120.4
O7—Ni1—O6^i^	90.23 (18)	C1—C6—H6	120.4
O6—Ni1—O6^i^	180.000 (3)	N1—C7—C8	121.5 (6)
C7—N1—C4	120.7 (5)	N1—C7—H7	119.3
C9—O4—H4	109.5	C8—C7—H7	119.3
Ni1—O5—H5A	112.9	C9—C8—C13	119.8 (6)
Ni1—O5—H5B	112.8	C9—C8—C7	121.0 (6)
H5A—O5—H5B	110.6	C13—C8—C7	119.1 (6)
Ni1—O6—H6A	111.7	O4—C9—C8	122.0 (6)
Ni1—O6—H6B	111.9	O4—C9—C10	119.4 (6)
H6A—O6—H6B	109.6	C8—C9—C10	118.6 (7)
Ni1—O7—H7A	113.0	C11—C10—C9	121.4 (7)
Ni1—O7—H7B	112.9	C11—C10—H10	119.3
H7A—O7—H7B	110.5	C9—C10—H10	119.3
O3—S1—O1	111.7 (2)	C10—C11—C12	120.9 (7)
O3—S1—O2	112.3 (2)	C10—C11—H11	119.5
O1—S1—O2	112.5 (2)	C12—C11—H11	119.5
O3—S1—C1	106.6 (2)	C13—C12—C11	118.7 (8)
O1—S1—C1	107.3 (2)	C13—C12—H12	120.6
O2—S1—C1	105.9 (2)	C11—C12—H12	120.6
C6—C1—C2	120.8 (5)	C12—C13—C8	120.5 (7)
C6—C1—S1	119.0 (4)	C12—C13—H13	119.7
C2—C1—S1	120.2 (4)	C8—C13—H13	119.7
C3—C2—C1	119.0 (5)		
			
O3—S1—C1—C6	163.8 (4)	C2—C1—C6—C5	0.6 (8)
O1—S1—C1—C6	−76.3 (5)	S1—C1—C6—C5	177.7 (4)
O2—S1—C1—C6	44.1 (5)	C4—N1—C7—C8	−177.6 (5)
O3—S1—C1—C2	−19.0 (5)	N1—C7—C8—C9	3.2 (10)
O1—S1—C1—C2	100.8 (4)	N1—C7—C8—C13	−179.6 (6)
O2—S1—C1—C2	−138.8 (4)	C13—C8—C9—O4	179.1 (6)
C6—C1—C2—C3	−1.1 (8)	C7—C8—C9—O4	−3.8 (10)
S1—C1—C2—C3	−178.2 (4)	C13—C8—C9—C10	−0.4 (10)
C1—C2—C3—C4	0.8 (9)	C7—C8—C9—C10	176.8 (6)
C2—C3—C4—C5	−0.1 (10)	O4—C9—C10—C11	−179.3 (6)
C2—C3—C4—N1	178.9 (6)	C8—C9—C10—C11	0.2 (10)
C7—N1—C4—C3	29.9 (9)	C9—C10—C11—C12	1.3 (11)
C7—N1—C4—C5	−151.1 (6)	C10—C11—C12—C13	−2.4 (11)
C3—C4—C5—C6	−0.4 (10)	C11—C12—C13—C8	2.2 (10)
N1—C4—C5—C6	−179.5 (6)	C9—C8—C13—C12	−0.8 (10)
C4—C5—C6—C1	0.2 (9)	C7—C8—C13—C12	−178.0 (6)

Symmetry codes: (i) −*x*+1, −*y*+2, −*z*+2.

### Hydrogen-bond geometry (Å, °)

**Table:**

*D*—H···*A*	*D*—H	H···*A*	*D*···*A*	*D*—H···*A*
O4—H4···N1	0.82	1.86	2.595 (9)	148
O5—H5A···O1^ii^	0.85	1.96	2.737 (6)	151
O5—H5B···O2^iii^	0.85	1.98	2.751 (6)	150
O6—H6A···O3^ii^	0.85	1.95	2.764 (7)	161
O6—H6B···O1^iv^	0.85	1.97	2.768 (8)	156
O7—H7A···O2	0.85	2.00	2.757 (8)	148
O7—H7B···O3^iii^	0.85	2.00	2.769 (7)	150
C2—H2···O3	0.93	2.56	2.917 (7)	104

Symmetry codes: (ii) *x*, *y*, *z*+1; (iii) *x*−1, *y*, *z*; (iv) *x*+1, *y*, *z*+1.

## References

[bb1] Bruker (2000). *SMART*, *SAINT* and *SADABS* Bruker AXS Inc., Madison, Wisconsin, USA.

[bb2] Sheldrick, G. M. (2008). *Acta Cryst.* A**64**, 112–122. 10.1107/S0108767307043930 18156677

[bb3] Tai, X.-S. & Feng, Y.-M. (2008). *Acta Cryst.* E**64**, o707.

[bb4] Tai, X.-S., Feng, Y.-M. & Zhang, H.-X. (2008). *Acta Cryst.* E**64**, m502. 10.1107/S1600536808005060 PMC2960793 21201882

[bb5] Tai, X. S., Yin, J. & Feng, Y. M. (2007). *Z. Kristallogr. New Cryst. Struct.* **222**, 398–400.

[bb6] Tai, X. S., Yin, J., Feng, Y. M. & Kong, F. Y. (2007). *Chin. J. Inorg. Chem.* **23**, 1812–1814.

[bb7] Tai, X.-S., Yin, J. & Hao, M.-Y. (2007). *Acta Cryst.* E**63**, m1061–m1062.

[bb8] Tai, X.-S., Yin, X.-H., Tan, M.-Y. & Li, Y.-Z. (2003). *Acta Cryst.* E**59**, o681–o682.

[bb9] Wang, L.-H., Yin, J. & Tai, X.-S. (2007). *Acta Cryst.* E**63**, m1664.

